# Time factor adjustment and performance across race formats and contrasting courses in sit para-​biathlon

**DOI:** 10.1186/s13102-026-02236-0

**Published:** 2026-11-05

**Authors:** Gauthier Rispal, Marie Ohlsson, Peter Edholm, Craig A. Staunton

**Affiliations:** 1 Department of Health Sciences, Swedish Winter Sports Research Centre, Mid Sweden University, Östersund, Sweden; 2 Gauthier Sport Scientist, Lyon, France; 3 Department of Physiology, Nutrition and Biomechanics, The Swedish School of Health and Sport Sciences (GIH), Stockholm, Sweden; 4 Department of Environmental and Bioscience, School of Business, Innovation and Sustainability, Halmstad University, Kristian IV:s väg 3, Halmstad, 301 18 Sweden

**Keywords:** Functional classification, Pacing, Para-Nordic skiing, Performance variability, Sit-ski, Time factor

## Abstract

Class-specific time factors (TFs) are applied across para-biathlon race formats and course profiles, but their evaluation requires an understanding of the competitions in which they are used. We examined race-score variability, skiing contribution and lap-speed profiles across Sprint, Middle and Long events at the relatively flatter 2022 (WC22) and hillier 2023 (WC23) world championships. Analyses included all 147 starts from 42 sitting-class athletes (16 women, 26 men). Mixed-effects models accounted for repeated starts and sex; paired athlete-level resampling quantified uncertainty in raw-to-adjusted coefficients of variation (CVs). TF adjustment reduced CV in nine of twelve sex-by-format-by-championship comparisons by 1.61–3.62% points. Mean adjusted CV was higher at WC23 (9.57%) than WC22 (8.52%), although the observed pattern depended on format: adjusted CV was higher at WC23 in Sprint and Middle but lower in Long for both sexes. Skiing contribution was 2.31% points greater at WC23 (95% CI 0.96 to 3.65), and the model-estimated average first-to-final lap-speed increase was 4.84 points greater (2.97 to 6.71). NoTF and ModTF had similar estimated championship differences in pacing; LargeTF estimates were smaller and less precise. Interactions did not establish different championship or format responses between TF groups. Complete lap profiles differed between championships in all three formats. These findings show that race demands and the dispersion of adjusted results vary across the competition contexts in which common TFs are applied. These findings support evaluating TFs against race format, course demands and penalty conventions. The apparently smaller difference between championships in LargeTF athletes’ first-to-final lap-speed change also warrants further investigation.

## Introduction

Biathlon is a winter sport that combines cross-country (XC) skiing with precision rifle shooting, where athletes compete over a designated course of a set distance [1]. Since the 1988 Paralympic Winter Games in Innsbruck, biathlon has also been open to athletes with physical or visual impairments, a discipline known as para-biathlon. Para-biathlon includes adaptations to support competition between eligible athletes, including shorter skiing distances, a shooting distance of 10 m rather than 50 m, and rifles remaining at the shooting range [2].

As in able-bodied biathlon, para-biathletes compete in Sprint (6–7.5 km), Middle (10 km), and Long (12.5 km) races, which differ in the number of shooting bouts and the form of penalty for missed shots. Due to these differences, athletes are likely to adopt different pacing strategies between race formats to optimize performance [2, 3]. Prior research in both able-bodied [3, 4] and para-​biathlon [2] has shown that pacing can vary significantly across events. While pacing strategies in able-bodied biathlon are relatively well-understood [3–5], where top athletes typically use a so-called ‘J-shaped’ pacing pattern or adjust speed based on terrain, the evidence base for pacing in para-biathlon athletes remains limited, largely because these disciplines have received little research attention. Moreover, individual physical limitations might necessitate specific pacing adaptations (e.g., reduced upper-body muscle mass, impaired neuromuscular control of the trunk, or challenges with balance during downhill sections).

In attempt to ensure fair competition, para-biathletes are classified into functional groups that reflect the impact of impairment on performance. Classification is conducted by panels typically including medical classifiers, such as physiotherapists or physicians, and technical classifiers with sport-specific or biomechanical expertise. The process includes both medical assessment and sport-specific functional evaluation (e.g., skiing posture, trunk control) and may be supplemented by in-competition observation. Athletes with visual impairments are grouped into B1–B3, where B1 indicates very low or no vision and B3 represents higher visual acuity. Standing athletes are classified LW2–LW9, where LW denotes Locomotor Winter, covering a spectrum of arm and/or leg impairments. Sitting athletes fall into LW10–LW12, ranging from no functional trunk control (LW10) to nearly full trunk stability (LW12) [6, 7].

Among these, the sitting category presents a particularly unique performance context due to the specialized equipment (sit-skis) and the exclusive use of the double-poling (DP) technique for both propulsion and balance [8–10]. These athletes face additional biomechanical and physiological constraints, including reduced trunk muscle control and upper-body power output, which directly affect pacing, fatigue, and race strategy. While the sitting category has attracted growing scientific interest [2, 7, 11], detailed analyses of performance across race formats and course conditions remain scarce.

Para-biathlon and para-Nordic skiing use a time factor (TF) system to allow athletes from different classifications to compete in the same event. The TF is a percentage-based adjustment applied to raw race times and is intended to compensate for impairment-related differences in performance [6, 7]. The practical question is whether a common class-specific adjustment remains appropriate across events with different performance demands. Changes in finishing-time dispersion provide one way to describe the adjustment, but the composition of race time and the development of skiing speed provide additional information about the competitions to which it is applied.

TF values reflect historical performance differences between classifications, with the same class-specific percentage applied across race formats and course profiles. Sprint, Middle and Long differ in skiing distance, shooting frequency and penalty structure, while courses differ in climbing demands. An adjustment derived from overall results may therefore be applied in settings with different combinations of skiing and shooting demands. This raises a practical question: do race-time composition and skiing-speed patterns vary across formats and competition environments in the same way for sitting-class groups receiving different TF adjustments? Examining these patterns provides a basis for evaluating the transferability of TFs across competition contexts.

Research in para-XC skiing has found broadly similar proportions of race time spent on different terrains across sitting classes [7]. Para-biathlon adds repeated shooting bouts and format-specific penalties, making race demands more complex. An earlier study of six sit para-biathletes described format-related differences in race-time composition and identified short uphill sections where skiing performance differentiated athletes [2]. A broader analysis combining raw and adjusted results, skiing contribution and complete lap profiles can extend this evidence across functional groups, women and men, and contrasting championship environments. These complementary outcomes describe how results are adjusted and how the races unfold; the lap profiles are not themselves altered by multiplying all times by a constant TF.

Therefore, this exploratory study examined race-score variability and skiing performance among women and men in the sitting classes at two para-biathlon world championships with contrasting course profiles. We addressed three questions: (1) how does TF adjustment change race-score variability across formats and the relatively flatter and hillier competition environments; (2) do format-related differences in skiing contribution vary among functional groups; and (3) do championship-related differences in lap-speed change vary among functional groups? We compared groups receiving no, moderate and larger TF adjustments across Sprint, Middle and Long, combining direct interaction tests with the magnitude and uncertainty of group-specific estimates.

## Methods

### Participants and race timing data

Race timing records from the sitting classes at the 2022 World Para Snow Sports Championships (Lillehammer, Norway; WC22) and the 2023 championships (Östersund, Sweden; WC23) were analysed. The processed dataset contained 147 athlete starts across six championship-by-format combinations and twelve sex-specific race cells (Table 1). An athlete start denotes one athlete’s participation in one race. Official timing records were originally obtained from EliteTiming AB (Sweden) and the International Biathlon Union’s public data centre (http://www.biathlonresults.com).

**Table 1:** Athlete starts by format, functional group, sex and championship in the primary analysis

	WC22		WC23	
	Women	Men	Women	Men
Long	12	19	4	13
NoTF	8	11	1	7
ModTF	1	6	2	4
LargeTF	3	2	1	2
Middle	12	18	5	12
NoTF	8	11	0	7
ModTF	1	6	3	4
LargeTF	3	1	2	1
Sprint	11	21	6	14
NoTF	8	12	1	7
ModTF	0	6	3	5
LargeTF	3	3	2	2

Entries are starts. LargeTF = LW10/LW10.5; ModTF = LW11/LW11.5; NoTF = LW12. Women contributed 26 NoTF, 10 ModTF and 14 LargeTF starts from 9, 3 and 4 athletes, respectively; men contributed 55, 31 and 11 starts from 15, 7 and 4 athletes. WC22 = Norway 2022 (relatively flatter); WC23 = Sweden 2023 (hillier). Long denotes the 12.5 km Individual format

The sample comprised 50 starts from 16 women and 97 starts from 26 men. Eleven athletes appeared at both championships. Two athletes contributed one start, four contributed two, 26 contributed three, one contributed five, and nine contributed six. Counts of unique athletes in LW10, LW10.5, LW11, LW11.5 and LW12 were 2, 3, 2, 1 and 9 for women, and 1, 3, 1, 6 and 15 for men, respectively. One woman appeared in both LW10 and LW10.5, so the female class counts sum to more than the number of distinct women.

### Study design and sample size

This study used a fixed dataset from two championships and was exploratory [12]. No prospective sample-size calculation determined recruitment. The 147 starts came from 42 distinct athletes; repeated starts were accounted for in the models, and precision was described using confidence intervals.

### Procedures

**Fig. 1:**
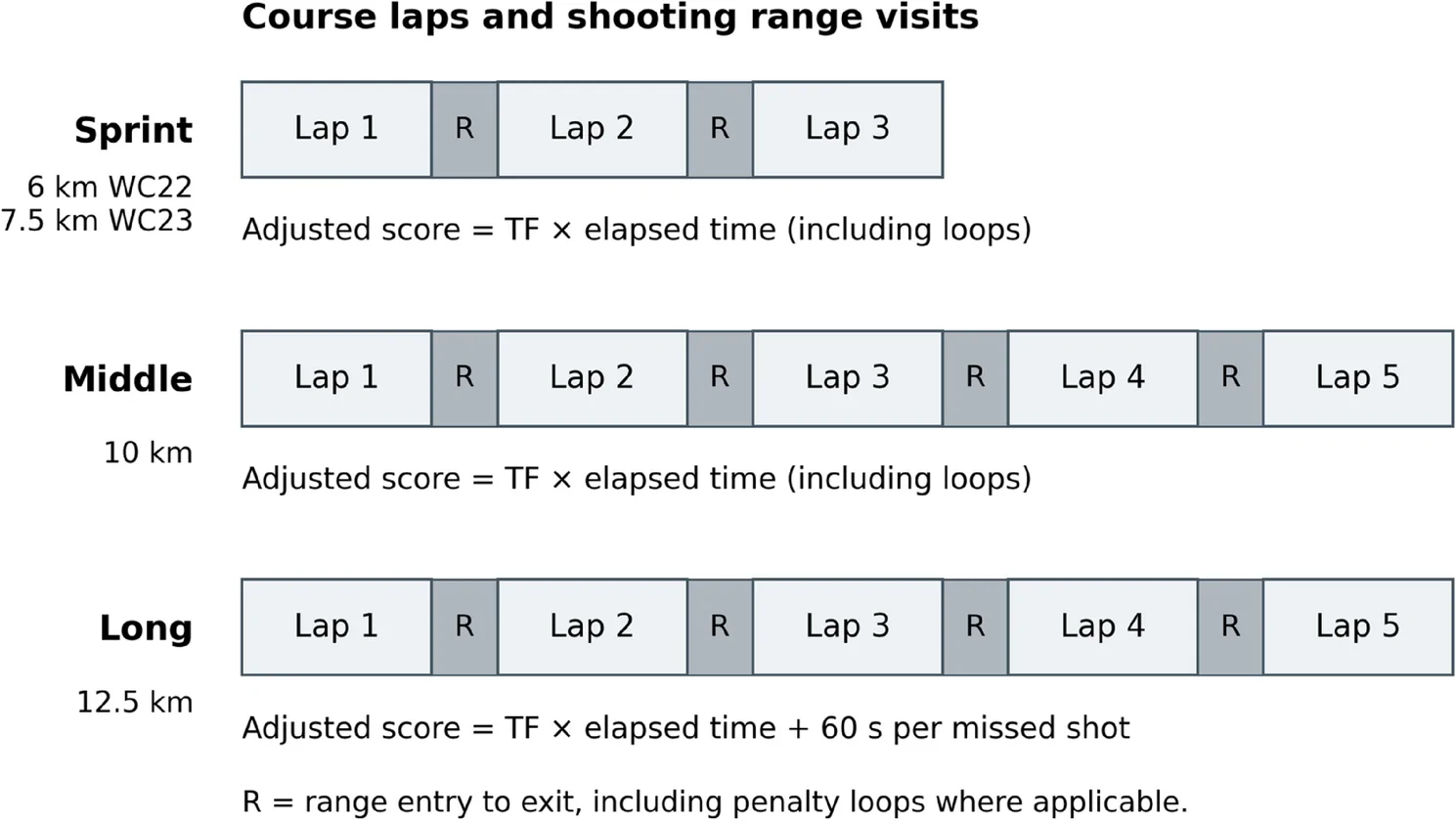
Race sequence and timing intervals in Sprint, Middle and Long formats. R denotes the interval between range entry and exit; it includes penalty-loop time in Sprint and Middle. Course laps exclude this entire interval. Sprint comprised three laps and two range intervals; Middle and Long comprised five laps and four intervals. Elapsed time, including loops, receives TF in Sprint and Middle. In Long (Individual), fixed penalties of 60 s per missed shot are added after TF adjustment. The sequence is schematic and not drawn to scale

All sitting-class events for women and men at WC22 and WC23 were analysed. Races used individually staggered starts. Long (Individual) covered approximately 12.5 km over five laps with four shooting bouts; each missed shot added a fixed 60 s penalty after TF adjustment. Middle covered approximately 10 km over five laps with four shooting bouts. Sprint covered approximately 6 km at WC22 and 7.5 km at WC23 over three laps with two shooting bouts. In Sprint and Middle, each missed shot required a penalty loop; its duration was included in elapsed time before TF adjustment. Figure 1 summarises the sequence and scoring convention.

Rifles remained at the shooting range and athletes did not carry them while skiing. Competition procedures and penalty rules followed the FIS Para Nordic Rules and Regulations 2022/2023 (arts 12317.1.2 and 12330.5). Timing records marked entry to and exit from the range; Fig. 1 identifies the course-skiing and range intervals.

The courses differed in elevation profile (Table 2). WC23 had greater height differences and maximum climbs, and included A climbs in the sit-ski homologation classification. A and B denote the climbing categories described in the FIS Para Nordic Homologation Guide (November 2023).

**Table 2:** Course elevation profiles at the relatively flatter WC22 and hillier WC23

	WC22						WC23					
	Distance per lap (m)	Height difference (m)	Max Climb (m)	Total Climb (m)	A climbs	B climbs	Distance per lap (m)	Height difference (m)	Max Climb (m)	Total Climb (m)	A climbs	B climbs
Sprint	2122	10	8	35	0	2	2410	28	12	44	2	2
Middle	2122	10	8	35	0	2	2020	19	12	33	1	2
Long	2691	12	8	47	0	2	2410	28	12	44	2	2

A and B denote sit-ski homologation climbing categories; total climb is accumulated ascent per lap

WC22 and WC23 are described as the relatively flatter and hillier competition environments, respectively. These labels refer to the measured course profiles; the comparison also includes differences in venue, year, participating field and conditions, and Sprint distance differed between championships.

### Time factor groups

Adjacent official sitting classes were combined into three analytical groups: LargeTF comprised LW10 and LW10.5 (TF 86% and 87%), ModTF comprised LW11 and LW11.5 (93% and 96%), and NoTF comprised LW12 (100%). Combining adjacent classes supported comparisons within the small field while retaining the functional distinctions represented by the official classes. Each start’s original LW class and exact TF percentage were retained. One athlete changed from LW10.5 to LW10 between championships, remaining in LargeTF. Group comparisons describe athletes with different functional characteristics and do not isolate an effect of applying TF.

### Data analysis

Race timing data included raw and TF-adjusted race times, course-lap times and distances, and timing splits recorded at entry to and exit from the shooting range. Skiing time was calculated by summing the three Sprint or five Middle/Long course-lap durations and excluded the complete range interval. In Sprint and Middle, this interval included any penalty loops, and TF adjustment was applied to the total elapsed race time. In Long (Individual), a fixed penalty of 60 s per missed shot was added after TF adjustment to determine the final race score. Range time and penalty-loop duration were not analysed as separate outcomes.

Lap skiing speed was calculated by dividing the recorded course-lap distance by its raw duration. Recorded split distances were 1.9, 2.0, 2.4–2.5 km. Skiing contribution was skiing time divided by the final race score, including all penalties, and multiplied by 100. Raw scores included unadjusted elapsed time plus any fixed penalties; adjusted scores included TF-adjusted elapsed time plus the same fixed penalties. Adjusted times were recalculated from raw durations and each start’s assigned TF percentage. All calculations used unrounded times.

Pacing was described using the percentage change in skiing speed relative to the first lap. First-to-final lap-speed change summarised the change between the beginning and end of each race, while every intermediate lap was retained in the profile plots and lap-level models. Positive values indicated faster skiing relative to the first lap, and negative values indicated slower skiing. These measures describe speed changes rather than fatigue.

### Statistical analysis

The coefficient of variation (CV; standard deviation divided by the mean, multiplied by 100) described the spread of race scores within each sex, championship and format. Raw and TF-adjusted scores used the same starts and included all penalties. CV reduction was expressed as raw minus adjusted CV in percentage points. Championship averages gave equal weight to the six sex-by-format combinations. We calculated 95% confidence intervals using 2,000 bootstrap samples of athletes, separately for women and men. All starts from a sampled athlete were kept together, and raw and adjusted scores remained paired.

Linear mixed-effects models accounted for repeated starts by allowing each athlete a separate baseline outcome (random intercept). Models included sex, championship, format and TF group. The pacing model included all two-way interactions between championship, format and TF group; the skiing-contribution model included the format-by-group interaction. These interactions tested whether championship differences in pacing, or format differences in skiing contribution, varied between functional groups. Models were fitted using restricted maximum likelihood, sum-to-zero coding of categorical predictors and Satterthwaite degrees of freedom [13, 14].

Estimated comparisons gave equal weight to the other modelled categories. We compared championships within each TF group and format, and directly compared the championship differences between groups. We also compared formats and TF groups for skiing contribution. Holm adjustment was applied jointly to the two principal interaction tests and separately across each family of nine comparisons. Confidence intervals for individual comparisons were pointwise 95% intervals.

Complete lap profiles were analysed separately for Sprint, Middle and Long using log-transformed raw lap speed. Each model included lap, championship, TF group and sex, with lap-by-championship, lap-by-group and championship-by-group interactions. Random intercepts for athlete and race start accounted for repeated starts and repeated laps within a start. The six tests of lap-by-championship and lap-by-group interactions were Holm-adjusted together. All models adjusted for an average sex difference; the figures also present women and men separately.

Residual plots and model convergence were inspected, and all valid observations were retained. Statistical significance was assessed at α = 0.05, with emphasis on estimated differences and their uncertainty. Analyses used R 4.3.2–4.3.3 with lme4, lmerTest and emmeans.

## Results

### Time factor adjustment and the penalty convention

**Fig. 2:**
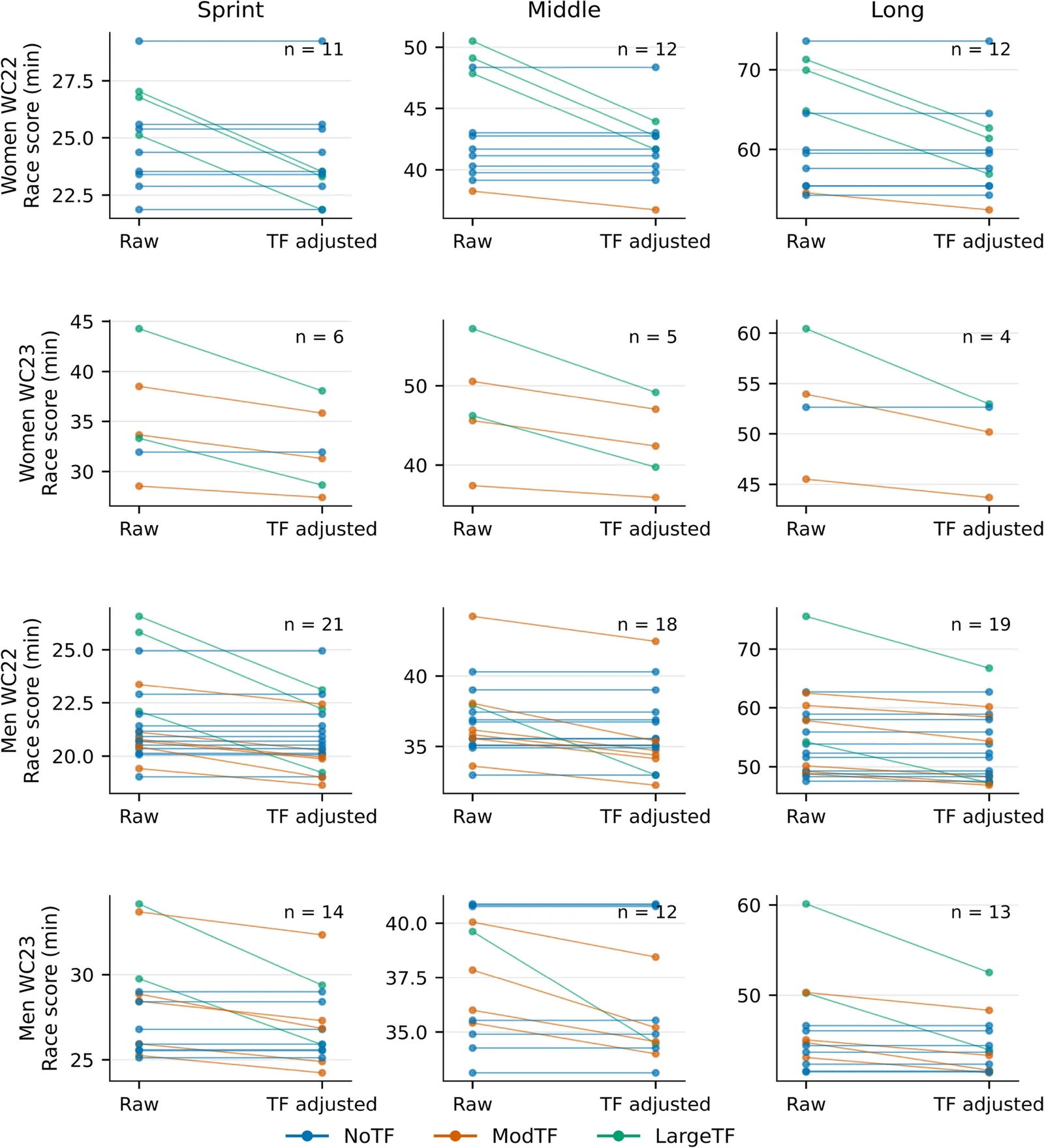
Paired raw and TF-adjusted race scores for the same starts, separated by sex, championship and format. Each line connects one start; colours identify functional groups. Scores include fixed penalties in Long and penalty-loop elapsed time in Sprint/Middle. n denotes starts. WC22 = Norway 2022; WC23 = Sweden 2023. NoTF = LW12; ModTF = LW11/LW11.5; LargeTF = LW10/LW10.5

Figure 2 shows the change from raw to TF-adjusted race scores for each start. In Individual races, TF reduced skiing and elapsed race times while the fixed penalty of one minute per missed shot remained unchanged. Consequently, fixed penalties made up a larger proportion of the adjusted score, and the skiing contribution was up to 1.49% points lower than its raw-score equivalent. Skiing contribution was unchanged when no fixed penalties were incurred or no TF adjustment was applied. In Sprint and Middle, skiing and total elapsed time, including penalty loops, received the same adjustment, leaving skiing contribution unchanged.

### Coefficient of variation (CV)

TF adjustment reduced race-score CV in nine of twelve sex-by-format-by-championship comparisons, by 1.61–3.62% points (Table 3). CV increased in women’s Sprint at WC22 (0.17% points) and men’s Middle at WC22 and WC23 (0.17 and 0.41 points). After adjustment, WC23 had higher observed CV than WC22 in Sprint and Middle for both sexes, but lower CV in Long for both sexes. Thus, the direction of the championship difference in adjusted variability depended on race format. The 95% confidence intervals for all twelve individual CV reductions included zero.

**Table 3:** Race-score CV before and after TF adjustment across sex, format and championship

Sex	Format	Championship	*n*	Raw CV (%)	Adjusted CV (%)	CV reduction pp (95% CI)
Women	Sprint	WC22	11	8.49	8.67	-0.17 (-2.72 to 3.64)
Women	Sprint	WC23	6	15.83	12.73	3.10 (-2.92 to 6.57)
Women	Middle	WC22	12	9.89	6.86	3.02 (-0.28 to 6.23)
Women	Middle	WC23	5	15.28	12.52	2.76 (-3.42 to 6.00)
Women	Long	WC22	12	11.22	9.59	1.63 (-0.86 to 5.36)
Women	Long	WC23	4	11.51	8.61	2.90 (-2.48 to 7.80)
Men	Sprint	WC22	21	9.36	7.56	1.80 (-1.16 to 4.56)
Men	Sprint	WC23	14	10.52	8.12	2.40 (-0.09 to 5.17)
Men	Middle	WC22	18	7.10	7.27	-0.17 (-1.46 to 0.91)
Men	Middle	WC23	12	7.68	8.09	-0.41 (-2.13 to 1.86)
Men	Long	WC22	19	12.75	11.14	1.61 (-1.15 to 4.12)
Men	Long	WC23	13	10.95	7.33	3.62 (-0.31 to 5.64)

CV reduction = raw CV minus adjusted CV, in percentage points; positive values indicate reduced dispersion

Scores include fixed Individual penalties. Intervals are pointwise percentile 95% intervals from paired athlete-level bootstrap resampling, stratified by sex; *n* denotes starts. Point estimates use unrounded inputs

Mean CV across the six sex-by-format combinations decreased from 9.80% to 8.52% at the relatively flatter WC22 and from 11.96% to 9.57% at the hillier WC23. Mean reductions were 1.29% points (95% CI − 0.21 to 3.08) and 2.39 points (0.07 to 3.66), respectively. WC23 therefore showed both a larger observed mean reduction and greater remaining dispersion. The estimated between-championship difference in mean reduction was 1.11% points (95% CI − 1.81 to 2.90).

### Skiing contribution across formats and championships

**Fig. 3:**
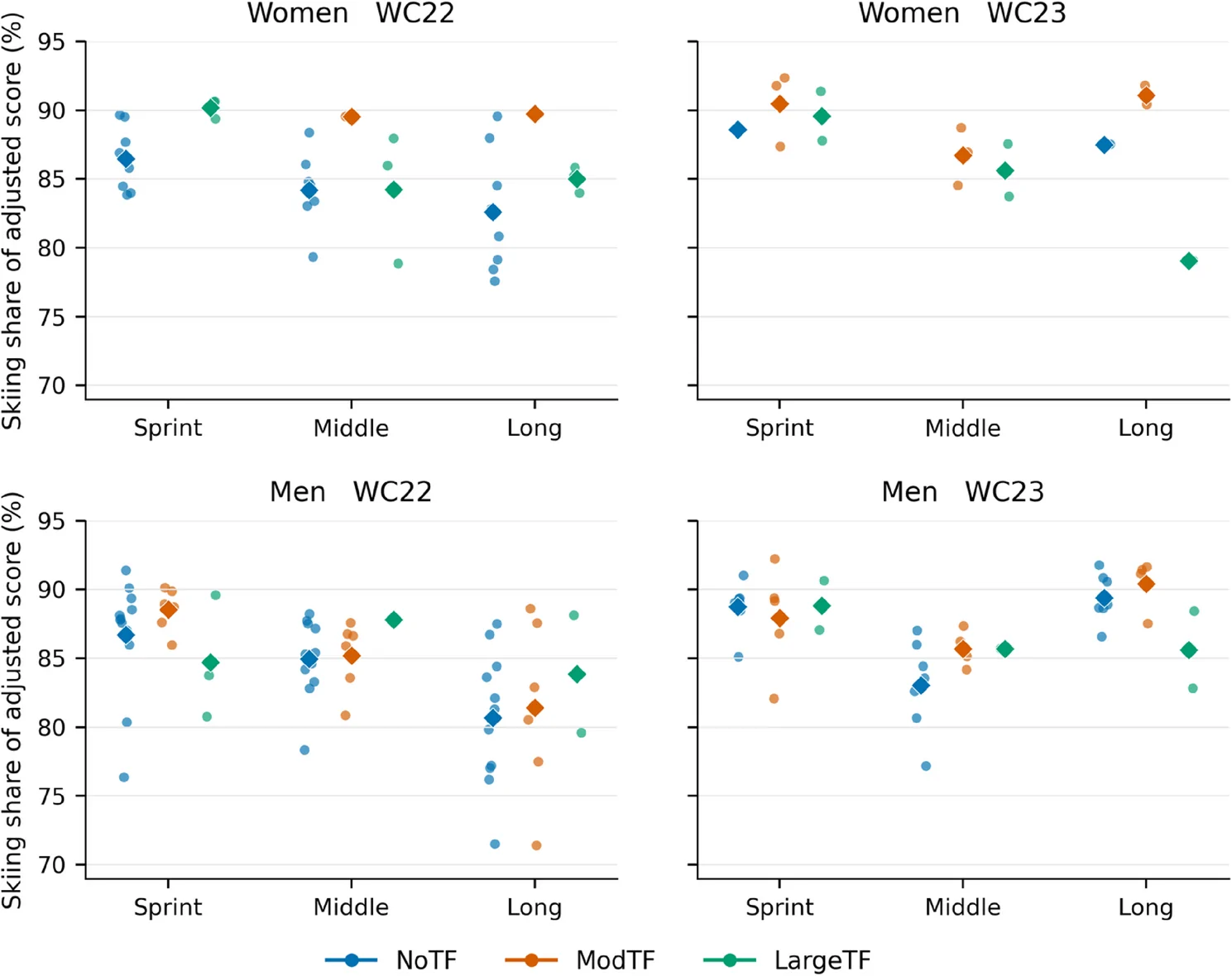
Individual observations of skiing time as a percentage of adjusted race score by sex, championship, format and functional group. Small circles denote starts and diamonds arithmetic means. All 147 starts are retained. Course skiing excludes the entire range interval, including penalty loops in Sprint and Middle; Long scores include fixed penalties added after TF adjustment. WC22 = Norway 2022 (relatively flatter); WC23 = Sweden 2023 (hillier). NoTF = LW12; ModTF = LW11/LW11.5; LargeTF = LW10/LW10.5

Skiing represented 71.39–92.35% of adjusted race scores (Fig. 3). Skiing contribution differed across race formats (F(2, 100.16) = 12.71, *p* < 0.001). All three TF groups had higher estimated skiing contributions in Sprint than in Middle or Long (Table 4). In NoTF, Sprint exceeded Middle by 3.05% points (95% CI 1.24 to 4.86; Holm *p* = 0.010) and Long by 3.59 points (1.79 to 5.38; Holm *p* = 0.001). Corresponding estimates were 2.78 and 2.42 points in ModTF and 3.62 and 4.06 points in LargeTF, although these comparisons did not meet the Holm-adjusted threshold. The format-by-group interaction was not supported (F(4, 99.62) = 0.21, unadjusted *p* = 0.931; Holm *p* = 1.000), so the data did not establish a format pattern unique to NoTF. Skiing accounted for a greater proportion of the adjusted score at the hillier WC23 than at the relatively flatter WC22 (estimated difference 2.31% points, 95% CI 0.96 to 3.65; F(1, 132.68) = 11.55, *p* = 0.0009), after accounting for format, TF group, sex and repeated starts.

**Table 4:** Estimated differences in skiing contribution between race formats within each functional group

TF group	Format comparison	Difference (pp)	95% CI (pp)	Holm *p*
NoTF	Sprint − Middle	3.05	1.24 to 4.86	0.010
NoTF	Sprint − Long	3.59	1.79 to 5.38	0.001
NoTF	Middle − Long	0.54	-1.29 to 2.36	1.000
ModTF	Sprint − Middle	2.78	0.26 to 5.31	0.186
ModTF	Sprint − Long	2.42	-0.16 to 5.00	0.263
ModTF	Middle − Long	-0.36	-2.93 to 2.20	1.000
LargeTF	Sprint − Middle	3.62	0.28 to 6.97	0.186
LargeTF	Sprint − Long	4.06	0.89 to 7.23	0.088
LargeTF	Middle − Long	0.44	-3.06 to 3.94	1.000

Differences are percentage points of the adjusted race score. Positive values indicate a greater skiing contribution in the first-named format. Estimates adjust for sex and championship and account for repeated starts. Intervals are pointwise 95% intervals; *p*-values are Holm-adjusted across all nine format comparisons. Consequently, an interval excluding zero need not correspond to an adjusted *p*-value below 0.05

### Endpoint pacing and complete lap profiles

Most starts showed a faster final lap than first lap: first-to-final lap-speed change ranged from − 18.88% to 16.41%, with positive changes in 136 starts and negative changes in 11. After accounting for sex and repeated athlete starts, the model-estimated average first-to-final increase was 4.84% points greater at the hillier WC23 than at the relatively flatter WC22 (95% CI 2.97 to 6.71; F(1, 122.51) = 26.35, *p* < 0.001).

Estimated WC23-minus-WC22 differences were positive in all nine TF-group-by-format combinations (Table 5). Estimates were similar in NoTF (4.50–6.21% points) and ModTF (4.47–6.17 points), with smaller and less precise estimates in LargeTF (3.06–4.77 points). After Holm adjustment, championship differences were supported for NoTF in all three formats and for ModTF in Middle and Long; none met the adjusted threshold in LargeTF. For Sprint, however, NoTF and ModTF estimates were almost identical (4.50 and 4.47 points), despite their different adjusted p-values. The championship-by-group interaction was not supported (F(2, 123.40) = 0.20, unadjusted *p* = 0.819; Holm *p* = 1.000), and no direct comparison of championship effects between groups was supported after adjustment. The estimated increase in speed from the first to the final lap differed less between championships in LargeTF than in NoTF or ModTF. However, uncertainty around these estimates and the unsupported interaction mean that this apparent group difference remains inconclusive.

**Table 5:** Estimated WC23-minus-WC22 differences in first-to-final lap-speed change within each functional group and race format

TF group	Race format	Difference (pp)	95% CI (pp)	Holm *p*
NoTF	Sprint	4.50	1.19 to 7.82	0.041
ModTF	Sprint	4.47	0.72 to 8.22	0.080
LargeTF	Sprint	3.06	-1.44 to 7.56	0.223
NoTF	Middle	5.29	1.82 to 8.75	0.022
ModTF	Middle	5.25	1.51 to 8.99	0.038
LargeTF	Middle	3.84	-0.90 to 8.59	0.223
NoTF	Long	6.21	2.83 to 9.59	0.004
ModTF	Long	6.17	2.39 to 9.95	0.013
LargeTF	Long	4.77	0.09 to 9.45	0.138

Positive differences indicate a larger first-to-final lap-speed increase at the hillier WC23 than at the relatively flatter WC22. Estimates adjust for sex and account for repeated starts. Intervals are pointwise 95% intervals; *p*-values are Holm-adjusted across all nine championship comparisons. The model estimates a common difference between groups’ championship effects across formats, so repeated differences between groups are not independent replications

**Fig. 4:**
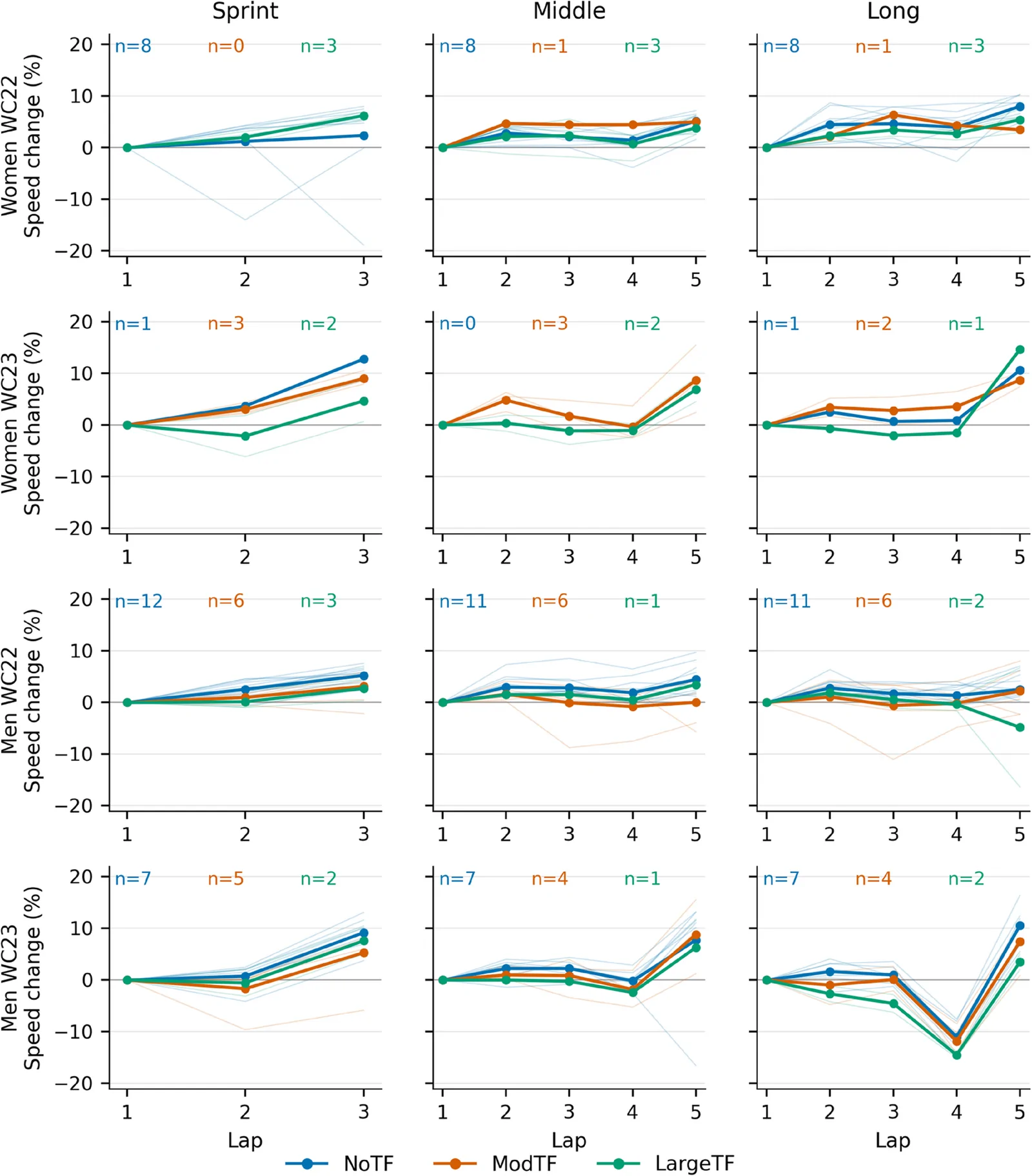
Complete lap-speed profiles for women and men at both championships. Speed change is relative to each start’s first lap. Faint lines represent starts and bold lines group means. Coloured panel counts denote starts per group. Sprint contains three laps; Middle and Long contain five. Raw lap times and recorded split distances were used. WC22 was the relatively flatter and WC23 the hillier competition environment. Negative changes are retained; uniform TF scaling leaves the relative profiles unchanged

The complete lap profiles showed that a faster final lap did not necessarily follow a steady increase in speed (Fig. 4). In particular, the men’s WC23 Long profiles showed a pronounced reduction on lap four followed by an increase on the final lap. Models incorporating every lap identified different patterns of speed change between championships in Sprint (F(2, 96) = 9.37), Middle (F(4, 172) = 8.21) and Long (F(4, 176) = 47.15; all Holm *p* < 0.001). Lap-by-group interactions were not supported in any format (all Holm *p* = 1.000). The figures describe the individual and group patterns separately for women and men; the fitted models estimated average sex-adjusted associations.

## Discussion

This study extends the limited evidence on sit para-biathlon by examining all recorded starts across three formats, two contrasting championship environments and the full sitting-class field. Three findings are particularly relevant to the evaluation of TFs. First, adjustment compressed most race-score distributions, but the remaining variability differed by format and championship. Second, skiing contribution and the first-to-final increase in lap speed were greater at the hillier WC23 than the relatively flatter WC22. Third, NoTF and ModTF showed similar estimated championship differences in pacing, while LargeTF estimates were smaller and less precise. Together, these results show why the performance contexts in which a TF is applied deserve attention alongside the final adjusted result.

### Time factor adjustment across competition contexts

The reduction in CV in nine of twelve race cells demonstrates that TFs generally narrowed the spread of scores in these championship fields. However, the larger observed mean reduction at WC23 coexisted with greater remaining adjusted dispersion. These findings answer different questions: how much the adjustment changes the field and how dispersed the adjusted field remains. Previous analyses of para-cross-country skiing have also used raw and adjusted results to characterise competitive differences, while work in elite skiing and biathlon shows that performance variability changes with the competition setting [15–17]. Consequently, a TF should not be judged solely by its ability to bring one field’s results closer together. Evaluation must also examine the relationship between impairment and performance, as required by evidence-based classification [6].

The format-specific pattern adds an important qualification to the terrain comparison. Adjusted CV was higher at WC23 in Sprint and Middle for both sexes, but lower in Long for both sexes. The hillier championship therefore did not produce uniformly greater dispersion. Differences in race duration, shooting frequency and penalty structure may contribute to this pattern, alongside the athletes and conditions in each field. This observation strengthens the case for examining competition formats separately when evaluating TF performance; a championship-wide average can conceal changes in opposite directions.

Skiing occupied most of the adjusted race score, but its relative contribution varied across formats. The higher Sprint estimates were present in all three functional groups, including LargeTF, whose Sprint-minus-Long estimate was numerically larger than NoTF’s. The supported NoTF comparisons therefore illustrate a broader pattern rather than a skiing-dominated format unique to that group. Earlier work in six sit para-biathletes likewise reported a lower mean skiing share in Long than Sprint and Middle, with a greater penalty contribution in Long [2]. Studies in able-bodied biathlon also show that the contributions of skiing and penalties to performance differences depend on event format [18–20]. Our time shares describe race composition, rather than the contribution of each component to differences between athletes, but both lines of evidence emphasise the importance of event structure.

The greater skiing contribution and larger first-to-final increase at WC23 are consistent with competition demands varying between relatively flatter and hillier environments. A mechanical basis for considering terrain is well established: uphill sit-skiing changes the balance between gravitational resistance, propulsion and technique, and sitting position influences performance [8–10]. In sit para-biathlon, short uphill sections have been associated with substantial differences in skiing time [2]. These studies make a terrain-related contribution to our championship patterns plausible. They do not determine how much of the present difference arose from terrain or whether its influence differed by class, particularly because some underlying mechanistic studies used able-bodied participants or a single athlete.

Evidence from terrain-specific race analysis also tempers a simple class-by-terrain explanation. Baumgart et al. found no clear class effect on the relative time spent in different terrains among sitting para-cross-country skiers, although performance rank and sex were associated with terrain-time distribution [7]. Thus, changes in overall course demand do not necessarily imply that class-specific factors must change. The present biathlon findings add the effects of shooting interruptions and penalty conventions to this discussion. Their contribution is to identify which aspects of performance differ across competition contexts and which apparent group patterns require a direct test, rather than to presume that every course difference creates a compensation problem.

The lap profiles provide a further contribution beyond finishing times. Most athletes completed the final course lap faster than the first, and the average increase was greater at WC23. However, complete profiles showed that this endpoint could follow intermediate slowing, especially the pronounced fourth-lap reduction among men in WC23 Long. A finishing-lap increase alone therefore gives an incomplete account of how the race unfolded. Research in biathlon and Nordic skiing similarly emphasises the influence of race structure, terrain and the distribution of speed or effort [3–5, 21]. For coaches, the practical implication is to inspect where speed is gained or lost across the whole race, using comparable course sections, rather than interpreting the final-lap increase alone as evidence of pacing quality or fatigue resistance.

The increase in speed from the first to the final lap was greater at the hillier WC23 than at the relatively flatter WC22 in all three functional groups. This championship difference was numerically smaller in LargeTF, suggesting that athletes with greater restrictions in trunk function may distribute their skiing speed differently as competition demands change. This is a relevant hypothesis because climbing demands and opportunities to increase speed may interact with the functional capacities available to the athlete. However, LargeTF included only eight athletes, its estimates were less precise, and direct comparisons did not establish different championship responses between groups. Further studies following the same athletes across contrasting courses are needed to determine whether this pattern reflects differences in how functional groups respond to course demands. Moreover, the pacing model estimated a common difference between groups’ championship effects across formats; the repeated separation between group estimates is not independent replication in three events. Event-dependent class patterns have been reported in para-cycling [22], supporting the broader rationale for examining impairment and event demands together. They provide a reason to test the hypothesis in sit para-biathlon, not evidence of a physiological ceiling in this sample.

The Individual scoring rule illustrates an additional reason why TF evaluation must account for race format. In Sprint and Middle, multiplying skiing and elapsed race time by the same factor preserves the skiing share. In Individual, fixed penalties are added after adjustment and therefore occupy a slightly larger share of the adjusted score for athletes receiving TF compensation. The observed reduction in skiing share, up to 1.49% points, follows directly from this convention. Thus, using the same TF percentage across formats does not give every component of the final score the same arithmetic treatment. This is not, by itself, evidence of unfairness; it identifies a scoring feature that should be explicitly considered when judging whether compensation operates as intended across biathlon formats.

Interpretation is limited by small and uneven groups, with only 11 of 42 athletes participating at both championships. Course profile coincided with venue and year, participating fields and snow conditions could differ, and Sprint distance changed. The models addressed repeated participation but could not separate these influences or establish causal terrain effects. They adjusted for an average sex difference, while sparse cells limited assessment of more complex sex-specific responses. Some large residuals also limit the precision of model-based inference. Course-lap speeds describe different segments of a race, and neither physiological capacity nor skiing economy was measured. Finally, the complete range interval included penalty loops in Sprint and Middle, so shooting execution and loop performance could not be examined separately. These considerations define the hypotheses that require confirmation without removing the observed differences in race composition and lap profiles.

### Implications for time factor evaluation and coaching

A common TF across formats is a practical competition rule, but its transferability should be evaluated rather than inferred from a reduction in finishing-time dispersion. Para-alpine skiing provides a precedent for discipline-specific factoring: separate factors are used for events such as slalom and downhill [23]. That precedent makes event-sensitive compensation a legitimate research question in para-biathlon, without establishing that new factors would improve its results. A defensible evaluation would follow the same athletes across multiple course profiles and formats, relate measured impairment to performance, and account for training and competitive level. The observed differences in race-score variability across formats and championships, together with the greater skiing contribution at WC23, identify relevant outcomes for such an evaluation. The pacing findings raise an additional question: do changes in course and competition demands influence the increase in speed from the first to the final lap differently across functional groups?

For athletes and coaches, the immediate application is to use format- and course-specific race profiles when reviewing performance. The predominance of skiing time supports attention to course performance, but the variation in skiing share cautions against assigning the same relative importance to skiing in every event. Individual lap profiles can identify unusually slow intermediate laps and place a fast finish in context. The modest change in skiing share caused by the Individual penalty convention should also be distinguished from an actual change in skiing behaviour. These applications can inform performance review now, while larger repeated-athlete studies address whether compensation should differ across events.

## Conclusion

TF adjustment narrowed race-score variability in most comparisons, but the remaining dispersion differed by format and championship. The hillier WC23 combined a greater skiing contribution with larger first-to-final lap-speed increases, while complete profiles revealed changes concealed by the endpoint summary. The difference between championships in the increase in speed from the first to the final lap was estimated to be smaller in LargeTF than in NoTF or ModTF. This numerical pattern warrants further investigation, although direct comparisons did not establish different championship responses between functional groups. These findings make a case for evaluating the common TF framework against the range of course demands, event structures and penalty conventions encountered in sit para-biathlon. Such evaluation should establish the conditions under which the existing factors perform adequately before deciding whether format-specific adjustments are warranted.

## Data Availability

The data from this study are publically available. Official timing data were obtained from EliteTiming AB (Sweden) and the International Biathlon Union’s public data centre (http://www.biathlonresults.com). All events were conducted in accordance with the rules of World Para Nordic Skiing (WPSN), under the governance of the International Paralympic Committee (IPC, 2020). The datasets used and/or analysed during the current study are available from the corresponding author on reasonable request.

## Abbreviations

ANOVA: Analysis of Variance
CV: Coefficient of Variation
DP: Double-poling
FIS: *Fédération Internationale de Ski* (International Ski and Snowboard Federation)
HD: Height Difference
IBU: International Biathlon Union
IPC: International Paralympic Committee
LargeTF: Large Time Factor group (TF 86–87%, classification LW10–10.5)
LW: *Locomotor Winter* (Prefix used for physical impairment classifications in Para-sports)
MC: Maximum Climb
ModTF: Moderate Time Factor group (TF 93–96%, classification LW11–11.5)
NoTF: No Time Factor group (TF 100%, classification LW12)
TC: Total Climb
TF: Time Factor
WC22: 2022 World Para Snow Sports Championships (Lillehammer, Norway)
WC23: 2023 World Para Snow Sports Championships (Östersund, Sweden)
WPSN: World Para Nordic Skiing
XC: Cross-country
